# Discovery That Theonellasterol a Marine Sponge Sterol Is a Highly Selective FXR Antagonist That Protects against Liver Injury in Cholestasis

**DOI:** 10.1371/journal.pone.0030443

**Published:** 2012-01-23

**Authors:** Barbara Renga, Andrea Mencarelli, Claudio D'Amore, Sabrina Cipriani, Maria Valeria D'Auria, Valentina Sepe, Maria Giovanna Chini, Maria Chiara Monti, Giuseppe Bifulco, Angela Zampella, Stefano Fiorucci

**Affiliations:** 1 Dipartimento di Medicina Clinica e Sperimentale, Nuova Facoltà di Medicina e Chirurgia, Università di Perugia, S. Andrea delle Fratte, Perugia, Italy; 2 Dipartimento di Chimica delle Sostanze Naturali, Università di Napoli, “Federico II”, Napoli, Italy; 3 Dipartimento di Scienze Farmaceutiche e Biomediche, Università di Salerno, Fisciano, Salerno, Italy; Clermont Université, France

## Abstract

**Background:**

The farnesoid-x-receptor (FXR) is a bile acid sensor expressed in the liver and gastrointestinal tract. Despite FXR ligands are under investigation for treatment of cholestasis, a biochemical condition occurring in a number of liver diseases for which available therapies are poorly effective, mice harboring a disrupted FXR are protected against liver injury caused by bile acid overload in rodent models of cholestasis. Theonellasterol is a 4-methylene-24-ethylsteroid isolated from the marine sponge *Theonella swinhoei*. Here, we have characterized the activity of this theonellasterol on FXR-regulated genes and biological functions.

**Principal Findings:**

Interrogation of HepG2 cells, a human hepatocyte cell line, by microarray analysis and transactivation assay shows that *theonellasterol* is a selective FXR antagonist, devoid of any agonistic or antagonistic activity on a number of human nuclear receptors including the vitamin D receptor, PPARs, PXR, LXRs, progesterone, estrogen, glucorticoid and thyroid receptors, among others. Exposure of HepG2 cells to theonellasterol antagonizes the effect of natural and synthetic FXR agonists on FXR-regulated genes, including SHP, OSTα, BSEP and MRP4. A proof-of-concept study carried out to investigate whether FXR antagonism rescues mice from liver injury caused by the ligation of the common bile duct, a model of obstructive cholestasis, demonstrated that theonellasterol attenuates injury caused by bile duct ligation as measured by assessing serum alanine aminostrasferase levels and extent of liver necrosis at histopathology. Analysis of genes involved in bile acid uptake and excretion by hepatocytes revealed that theonellasterol increases the liver expression of MRP4, a basolateral transporter that is negatively regulated by FXR. Administering bile duct ligated mice with an FXR agonist failed to rescue from liver injury and downregulated the expression of MRP4.

**Conclusions:**

FXR antagonism *in vivo* results in a positive modulation of MRP4 expression in the liver and is a feasible strategy to target obstructive cholestasis.

## Introduction

Cholestasis is a liver disorder that occurs primarily in the context of genetic mutation of basolateral or apical membrane transporters in hepatocytes. Cholestasis represents the main biochemical feature of primary biliary cirrhosis [1], [2] (PBC) and sclerosing cholangitis (PSC), two immune-mediated disorders characterized by progressive bile duct destruction for which medical therapy is still poorly effective and investigations are ongoing to identify novel therapeutic approaches [1], [2]. In addition to PSC and PBC, an obstructive form of cholestasis occurs in patients suffering from biliary stones or biliary and pancreatic tumors [1]. Theoretically, because PBC and PSC are characterized by bile duct destruction, therapy should be aimed at activating bile acid secretion from the basolateral membrane of hepatocytes, while stimulation of bile acid secretion from the apical membrane is likely to worsens liver injury due to the obstruction of bile flow [3]. FXR is a bile acid sensor that regulates bile acid synthesis and excretion. While activation of FXR favours bile acid detoxification by hepatocytes and FXR ligands have been proposed in the treatment of PBC patients [1], results from models of obstructive cholestasis in FXR^−/−^ mice have shown that FXR gene ablation protects against liver injury caused by ligation of common bile duct (BDL) [3]. Molecular decoding of the BDL model has lead to the demonstration that FXR functions as a negative regulator of multidrug resistance-associated protein (MRP)-4, a gene mediating basolateral secretion of bile acids. Thus, while FXR^−/−^ mice adapt to bile duct obstruction by an ≈20 fold induction in the expression of MRP-4 mRNA, these changes are not reproduced in wild type mice [3]. Because induction of MRP-4 represents an adaptive response to bile duct obstruction and protects the liver from accumulation of toxic bile acids during cholestasis by facilitating their efflux into blood for ultimate renal excretion, and MRP-4-knockout mice are sensitised to liver injury induced by BDL [4], regulation of this basolateral transporter exerts an essential role in orchestrating the adaptive changes under conditions of impaired bile flow due canalicular obstruction/destruction [2], [5]–[7]. In vitro characterization of interaction of FXR with MRP-4 has lead to the demonstration that FXR functions as a braking signals for MRP-4 induction caused by activation of Constitutive Androstane Receptor (CAR) [2], [5]–[7]. Gene promoter analysis of human MRP-4 promoter has revealed the presence of a CAR responsive element embedded within an FXR responsive element, an everted repeat (ER)-8, known to mediate repression of FXR target genes [5]. Thus, it appears that FXR competes with CAR for binding to this overlapping binding site and FXR ligation of ER-8 displaces CAR from the MRP-4 promoter abrogating MRP-4 induction caused by CAR activators [2], [5]. In aggregate, these data suggest that FXR activation in obstructive cholestasis might worsen liver injury by hijacking a protective mechanism regulated by CAR, i.e induction of MRP-4 [2], [6]–[8]. While these data strongly advocate the utility of an FXR antagonist in the treatment of obstructive cholestasis, this concept has remained unproved because the lack of a selective FXR antagonist [2].

The observation that ≈40% of modern pharmaceuticals are derived from biological sources [8]–[10], highlights the incredible biomedical potential represented by the chemical analysis of natural organisms [9], [10]. As the results of enzymatic reactions, natural products have an intrinsic capacity to recognize and bind macromolecules, perturb their activity, and modulate biological processes. Besides their potential use as pharmaceutical drugs, natural products have and will continue to play critical roles as biological probes, essential component of today's research arsenal and useful to dissect complex biological processes and ultimately, to identify novel therapeutic targets. Among natural sources, marine environment, with its vast pool of plants, animal and micro-organisms, represents a greater promise to provide original molecules for treatment of human diseases [9], [10]. Sponges of the genus *Theonella* have attracted the interest from the scientific community for the impressive variety of bioactive secondary metabolites with unusual structures and powerful biological effects [7]. Representative compounds include non-ribosomal peptides exemplified by the antifungal theonellamides, a new class of sterol-binding molecules that induce membrane damage and activate Rho1-mediated 1,3βD-glucan synthesis [11] and complex polyketides such as the actin-bounding macrolide swinholide A [12]. In addition, sponges of *Theonella* genus are distinctive in producing biosynthetically unique sterols [7]. Decodification of these non conventional steroids has allowed the identification of 24-ethylsterols endowed with potent activity towards mammalian nuclear receptors including the FXR and pregnane-X-receptor (PXR) [7], [13]. *Theonellasterol* is a 4-methylene-24-ethylsteroid that has been proposed as a taxonomic marker for the *Theonella* sponge phenotypes [14]. Structurally theonellasterol contains a relatively rare 8(14) double bond and a biosynthetically unusual 4-methylene functionality. This unusual functional group has been proposed to biogenetically arise from a shunt in the oxidative demethylation of the 4-methyl series, through the dehydration of the primary alcohol formed in the first oxidation of the methyl group [14]. The biological function of these 4-methylenesteroids is unknown. However, their relative abundance in the apolar extract of *Theonella swinhoei* as well as their ability to fit in the ligand binding domain of FXR and PXR [7], [14], emphasize a plausible role as putative ligands for ancestral sponge nuclear receptor(s).

In the present study, we report the discovery that *theonellasterol*, a 4-methylenesteroids isolated from the *Theonella swinhoei* sponge, is a highly selective FXR antagonist. Of interest, despite *theonellastero*l was known since long time (14), its effect on nuclear receptors has never been tested. Using this agent we have designed a proof-of-concept study to investigate whether pharmacological antagonism of FXR holds potential in protecting against liver injury in obstructive cholestasis [2], [5].

## Results

### Isolation of theonellasterol from *Theonella swinhoei*

**Figure 1:**
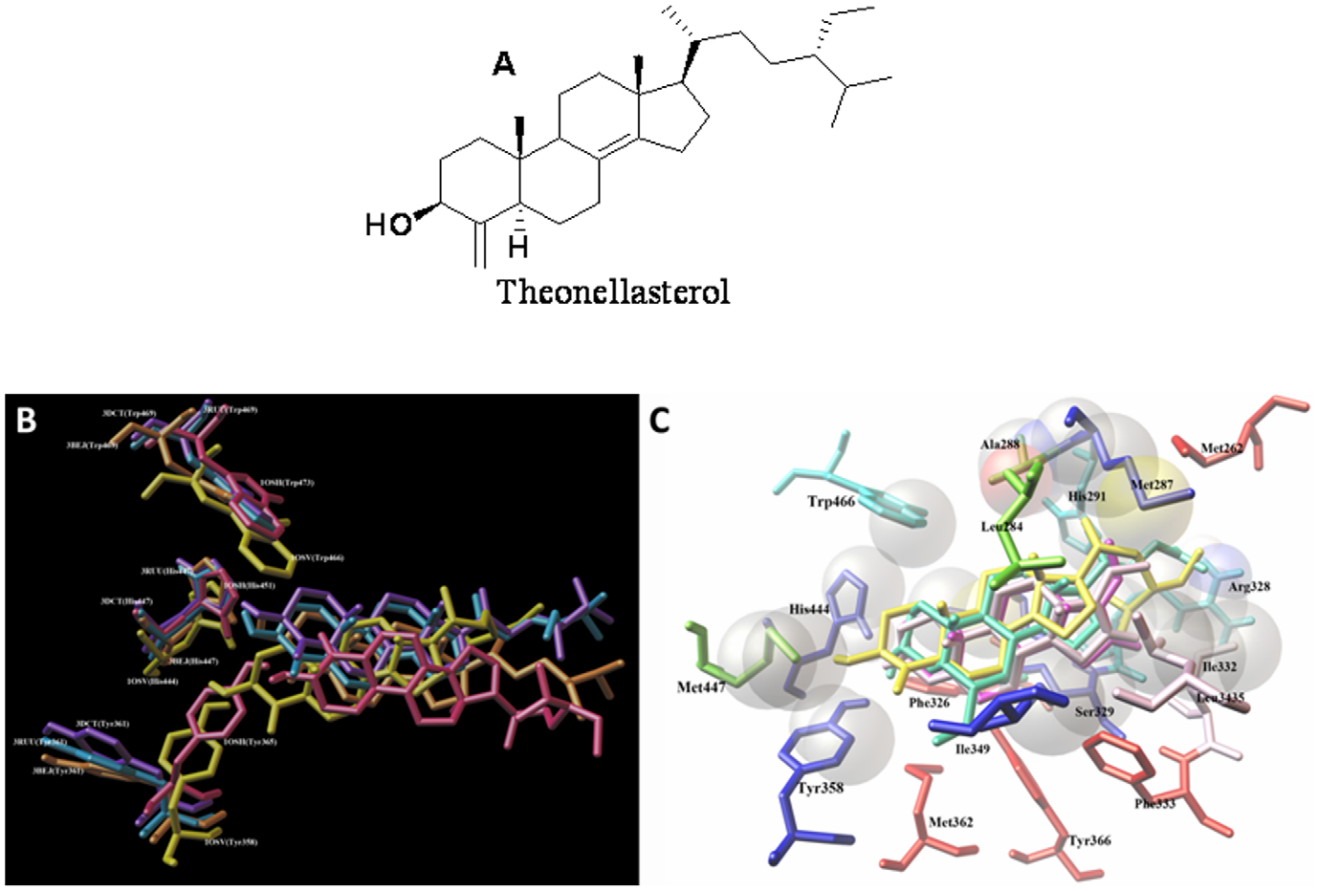
Theonellasterol from *Theonella swinhoei*. (A) Chemical structure of theonellasterol isolated from *Theonella swinhoei*. (B) Superimposition of the different docking poses of theonellasterol in the rat FXR (theonellasterol and 1OSV [20] yellow), and human FXRs (theonellasterol and 3BEJ [15] orange; theonellasterol and 1OSH [17] pink; theonellasterol and 3DCT [18] purple; theonellasterol and 3RUU [19] light blue). (C) Superimposition of theonellasterol (yellow) with 6-ECDCA (sky blue), and Z (pink)/ E (light pink) gugglusterone in the binding pocket of FXR (1OSV). Amino acids interacting with theonellasterol (yellow) are depicted in green, amino acids interacting with 6-ECDCA and theonellasterol are depicted in sky blue, amino acids interacting with Z/E gugglusterone and theonellasterol are depicted light pink, amino acids interacting with 6-ECDCA and Z/E gugglusterone in red, and amino acids interacting with all molecules are depicted in blue.

The initial processing of the *Theonella swinhoei* (coll. No. R3170) was conducted according to procedures described elsewhere [13]. The *n*-hexane extract from a solvent partitioning Kupchan procedure was cromatographed by silica gel and the fraction eluted with CH_2_Cl_2_∶MeOH 99∶1 was further purified by reverse phase HPLC to afford 40.5 mg of theonellasterol (Figure 1) as a colourless amorphous solid ([a]_D_^25^+20.4 *c* 1 MeOH). The identity of theonellasterol was secured by comparison of its NMR and MS spectrum (Table S1 and Figure S1) with those previously reported [14]. The purity was established major than 95% by HPLC trace (Figure S2).

### Docking Studies

The flexible nature of FXR side chains suggests that its ligand binding domain (LBD) may have considerable ability to accommodate differently shaped ligands changing its conformation in response to ligand binding [15]. In order to rationalize the binding mode of theonellasterol on FXR receptor, we have therefore performed docking calculations (by Autodock 4.2 software [16]) on several FXR structures co-crystallized with different compounds [17]–[20] with the aim to predict the position of the LBD in complex with theonellasterol. As reported, the activation of the FXR by the sterol molecules is, among the others, regulated by the interaction between the OH at C-3 of steroid skeleton and the amino acids of the catalytic triad (namely Tyr in Helix 7, His in Helix 10/11 and Trp in Helix 12) [20]. In all the three dimensional models (Figure 1B) theonellasterol interacts with the catalytic triad generating a hydrogen bonds with Tyr358 and 365 in the Helix 7 for the pdbs 1OSV [20] and 1OSH [17] respectively, and with His447 for the pdbs 3DCT [18], 3BEJ [15], 3RUU [19], and His444 for 1OSV [20] (Helix 10/11). On the other hand, only in the three dimensional models with 3DCT [18], 3RUU [19], and 1OSV [20] the marine sterol establishes hydrophobic interactions with Helix 12 and in particular with the Trp469 (3DCT [18], 3RUU [19]) or Trp466 (1OSV [20]) (Figure 1B). Considering also the additional interactions with the LBD, we have chosen the complex with the 1OSV [20] for our analysis because *theonellasterol* and 6-ECDCA show similar chemical features with respect to the other molecules co-crystallized with the FXR structures. On this basis, in addition to the two hydrogen bonds between theonellasterol with Tyr358 (Helix 7) and His444 (Helix 10/11) reported above, the *trans* junction between A/B rings and its peculiar unsaturation between C-8 and C-14 causes a different spatial arrangement with respect to the semi-synthetic agonist 6-ECDCA [20] and the natural antagonist guggulsterone (Figure 1C) [21], [22] isomers not allowing the hydrophobic contact with Met362, Phe326, Phe333 and Tyr366. On the other hand, theonellasterol's steroid skeleton interacts with Leu345 and Trp466 in the same manner that guggulsterone and 6-ECDCA respectively, and with Ala288, Leu284, and Met447; while its alkylic chains is in close contacts with Arg328 and His291, and with Ile332 (Figure 1C) as reported for 6-ECDCA and guggulsterone respectively. Furthermore, theonellasterol maintains the same hydrophobic interactions of 6-ECDCA and guggulsterone with His444, Ile349, Met287, Met325, Ser329, Tyr358 in the LBD. In conclusion, even if theonellasterol shows a relatively simple skeleton in comparison to the 4-methylene steroids previously described by us [13], docking results suggest that the different pattern of hydrophobic interactions established with FXR are sufficient to support its competition with 6-ECDCA in occupying the FXR binding site [5], [13], [20]–[23].

### Theonellasterol is an FXR antagonist and reverses the effect of CDCA on the expression of canonical FXR target genes

**Figure 2:**
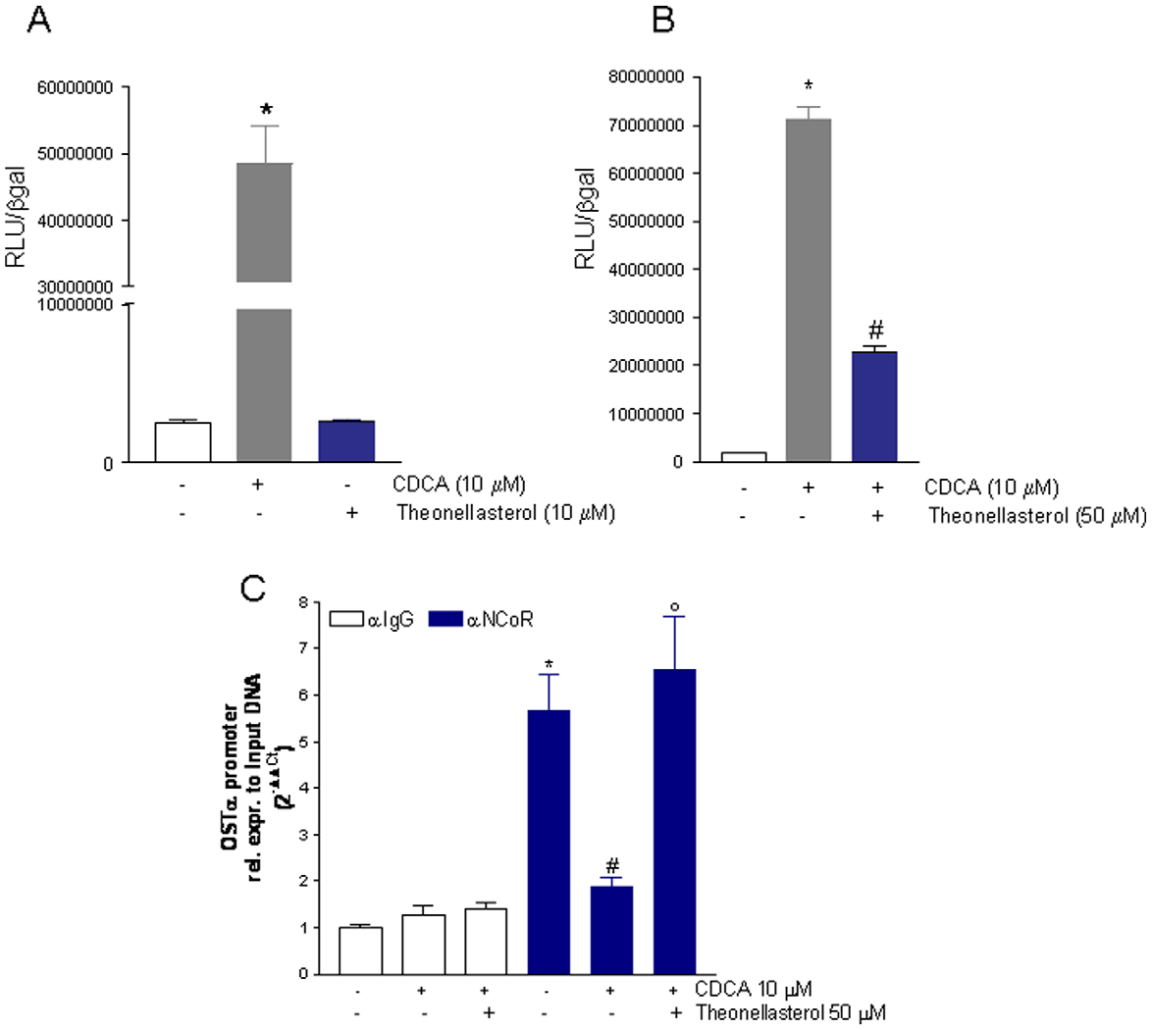
Theonellasterol is an FXR antagonist. Luciferase reporter assay performed in HepG2 transiently transfected with pSG5-PXR, pSG5-RXR, pCMV-βgal, pCYP3A4promoter-TKLuc vectors and stimulated 18 h with (A) 10 µM of CDCA or *theonellasterol* and (B) 10 µM of CDCA alone or in combination with *theonellasterol* 50 µM. Data are the mean ± S.E. of three experiments. *P<0.05 versus cells left untreated. #P<0.05 versus CDCA. (C) CHiP assay of NCoR binding to the OSTα promoter. CDCA displaces NCoR from OSTα and this effect is reversed by theonellasterol. RT-PCR analysis of proteins immune-precipitated with a control IgG are shown as control. Data are the mean ± S.E. of three experiments. *P<0.05 versus anti IgG immunoprecipitates. #P<0.05 CDCA versus cells left untreated; P<0.05 *thenollasterol* versus CDCA alone.

We have then investigated whether *theonellasterol* directly transactivates or inhibits FXR transactivation caused by CDCA, a canonical FXR ligand. For these purposes we used HepG2 cells, an hepatocarcinoma cell line transfected with FXR, RXR, β-galactosidase expression vectors (pSG5FXR; pSG5RXR and pCMV-βgal) and with a p(hsp27)TKLUC reporter vector containing the promoter of the FXR target gene heat shock protein 27 (hsp27) cloned upstream to the Luciferase gene. Twenty-four hour post-transfection, cells were challenged with CDCA, 10 µmol/l, *theonellasterol*, 10 µmol/l, or with the combination of the two (i.e. CDCA, 10 µmol/l, and *theonellasterol*, 50 µmol/l) for 18 h. As shown in Figure 2A and B, *theonellasterol* exerted no agonistic activity on the receptor but caused a robust attenuation of its transactivation induced by CDCA (n = 4; P<0.05 versus CDCA). Confirming its antagonistic activity on CDCA, exposure of HepG2 cells to 50 µM *theonellasterol* effectively stabilized the nuclear corepressor NCoR at its binding site in the promoter of OSTα, a well characterized FXR-regulated gene. Thus, as illustrated in Figure 2C, the ChIP analysis demonstrates that while exposure to CDCA, 10 µM, released NCoR from the OSTα promoter, co-treating cells challenged with CDCA with the *theonellastero*l, 50 µM, abrogated this pattern (n = 3; P<0.05 versed CDCA alone).

**Figure 3:**
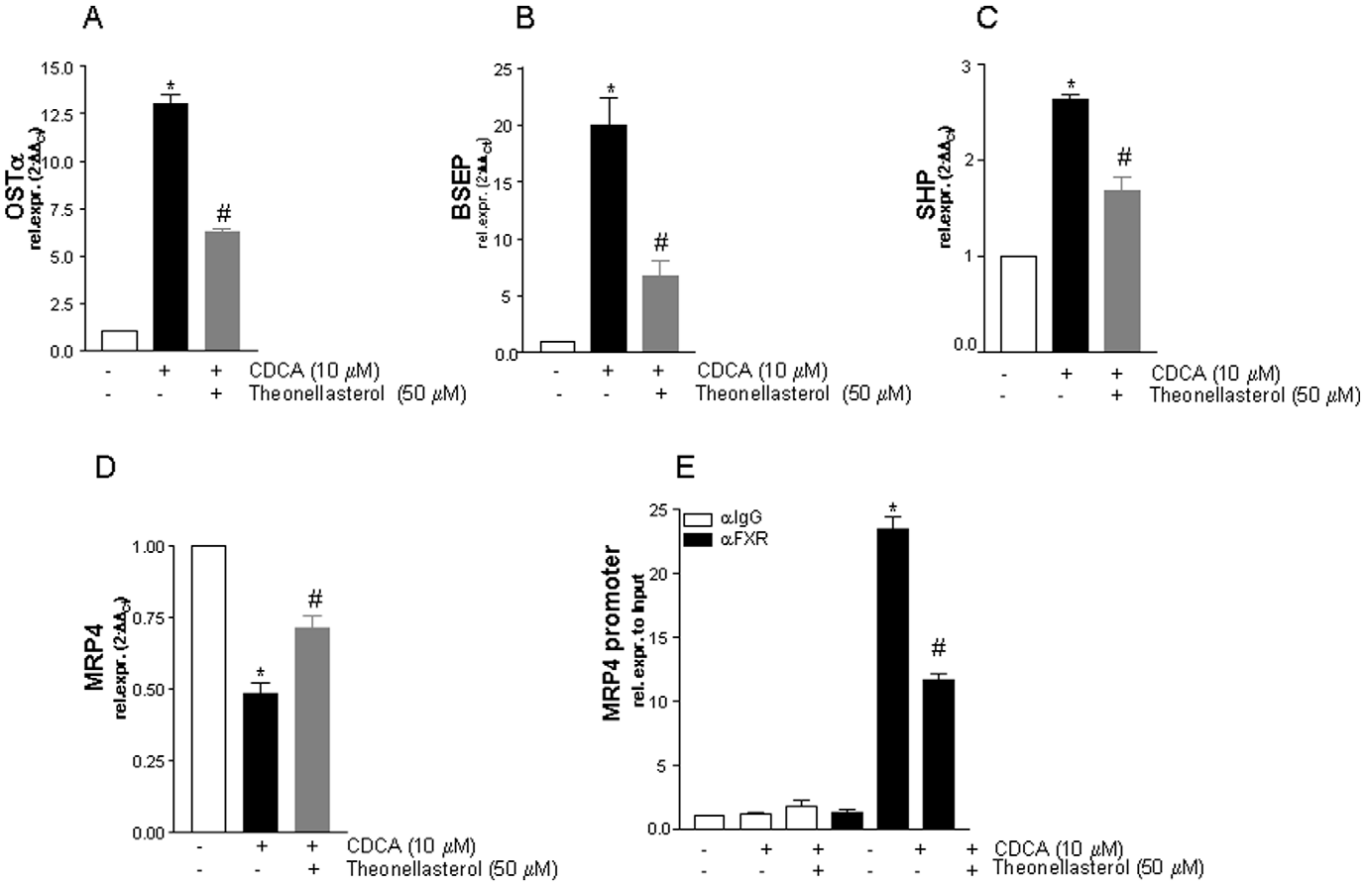
Theonellasterol reverses the effect of CDCA on the expression of canonical FXR target genes. Relative mRNA expression of (A) OSTα, (B) BSEP, (C) SHP and (D) MRP4 in HepG2 cells treated with 10 µM CDCA alone or with the combination of CDCA plus *theonellasterol* 50 µM. (E) CHiP assay performed in HepG2 cells not stimulated or primed with CDCA, 10 µM, alone or in combination with *theonellasterol*, 50 µM. Theonellasterol antagonizes the recruitment of FXR on the MRP4 promoter. Data are the mean ± S.E. of three experiments. *P<0.05 versus cells left untreated. #P<0.05 versus CDCA alone.

Because these data suggest that the *theonellasterol* was endowed with an FXR antagonistic activity, we have then tested its effects on the expression of known FXR target genes [2] using primary cultures of mouse hepatocytes. In this experimental setting, *theonellasterol* reversed the effect of CDCA on the expression of canonical FXR target genes [23]: OSTα, BSEP, and SHP (Figure 3A–C; n = 4; P<0.05 versus CDCA alone). Interestingly, the antagonistic activity of *theonellasterol* extended also to MRP-4 (Figure 3D) [2]–[5]. Because the regulatory activity of *theonellasterol* on MRP-4 holds promise for potential therapeutic use of this steroid in obstructive cholestasis, we have examined in a further detail the molecular mechanisms involved in this effect. More specifically, we have asked whether the antagonistic activity of *theonellasterol* on MRP-4 expression induced by CDCA was promoter specific. For this purpose a ChIP assay was carried by immune-precipitating nuclear extracts from HepG2 cells left untreated or primed with CDCA alone or with the combination of CDCA plus *theonellasterol* with an anti-FXR antibody. As shown in Figure 3E, results of Real-Time PCRs demonstrated that while in basal conditions, FXR is not constitutively bound to the MRP-4 promoter, but is recruited on the promoter following activation with CDCA. Recruitment of FXR to the MRP-4 promoter in the presence of CDCA was robustly attenuated by co-incubating the cells with the *theonellasterol* (n = 4; P<0.05 versus CDCA alone). All together these results indicate that *theonellasterol* exerts its antagonistic activity by reducing the binding of FXR on the MRP-4 promoter, thus preventing its down-regulation caused by CDCA (Figures 3D and E).

### *Theonellasterol* is a selective FXR antagonist

**Figure 4:**
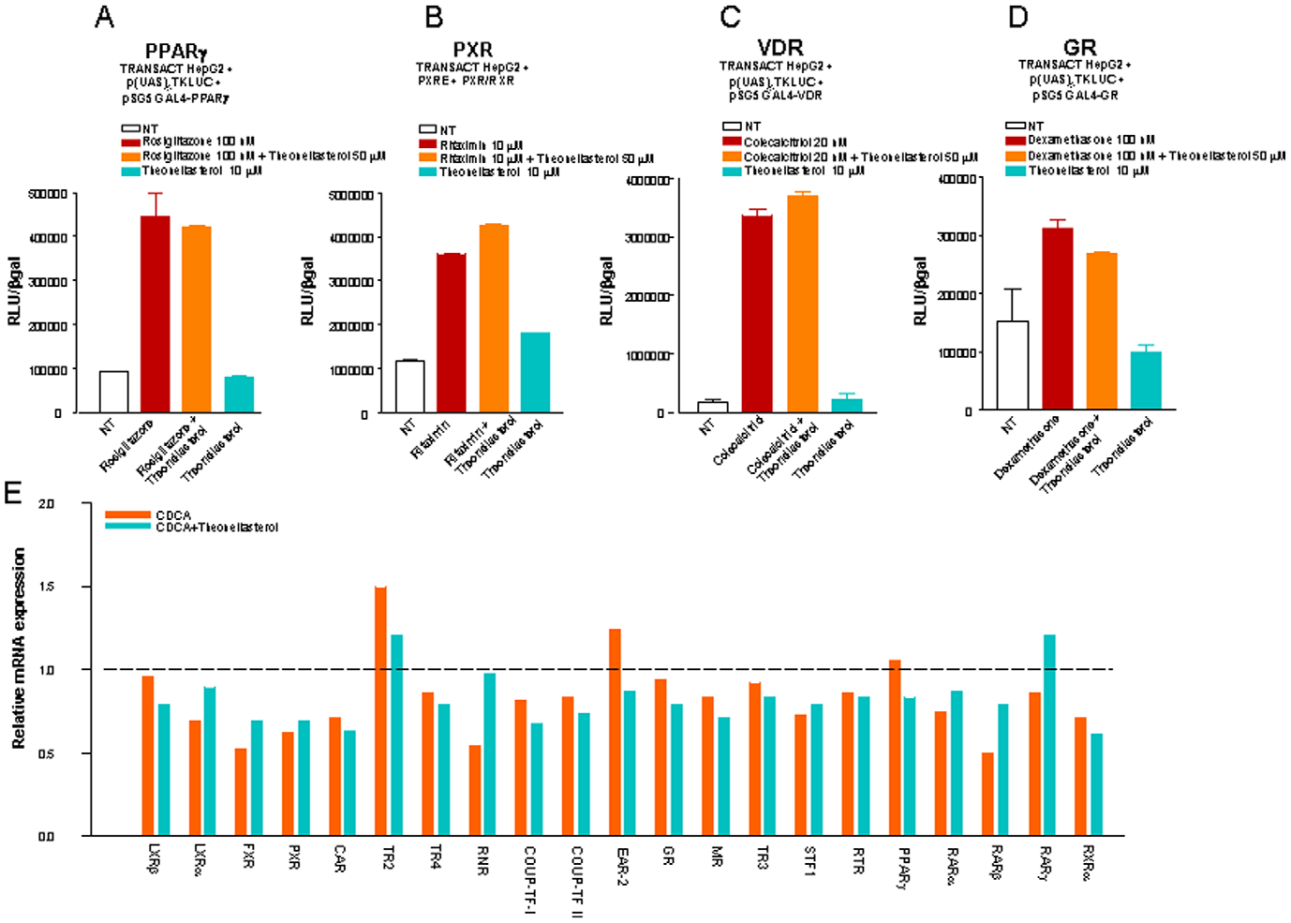
Theonellasterol is a selective FXR antagonist. (A) (C) and (D) HepG2 cells were co-transfected with the Gal4 luciferase reporter and a series of chimeras in which the Gal4 DNA binding domain is fused to the LBD of the indicated nuclear receptors. Cells were treated with the appropriate agonists or specific agonists in combination with *theonellasterol*. (B) HepG2 cells were co-transfected with pSG5-PXR, pSG5-RXR and with the reporter pCYP3A4promoter-TKLuc and then stimulated with rifaximin, a PXR agonist, alone or in combination with *theonellasterol*. Data are the mean ± S.E. of three experiments. *P<0.05 versus not treated cells. (E) Microarray analysis showing the relative mRNA expression of various nuclear receptors and nuclear receptors co-activators following stimulation of HepG2 cells with CDCA, 10 µM, alone or in combination with *theonellasterol*, 50 µM. Data are the mean ± S.E. of three experiments.

To further investigate the specificity of the above described interactions, we have tested whether *theonellasterol* interacts with other nuclear receptors including PXR, PPARγ, VDR and GR (i.e.receptors that are targeted by guggulsterone [21]–[23]). For this purpose we used fusions of the LBD of PPARγ, VDR and GR with a GAL4-DNA binding domain cloned into an expression vector (pSG5). Transactivation experiments were carried out using a reporter vector containing five repeats of the GAL4 responsive element cloned upstream the luciferase gene (p(UAS)_5x_-TK-Luc). To investigate the effect of *theonellastero*l on PXR, HepG2 cells were transfected with pSG5PXR, pSG5RXR, pCMV-βgal and with the reporter vector p(cyp3a4)TKLUC containing the PXR response element of Cyp3A4, a canonical PXR target gene, cloned upstream to the luciferase gene. As shown in Figure 4 A–D, *theonellasterol* at the concentration of 10 µM failed to transactivate PPARγ, PXR, VDR and GR, nor it inhibited the activation of these receptor promoted by specific ligands, i.e. rosiglitazone, rifaximin, 1,25 dihydroxy colecalciferol and dexamethasone when co-incubated with these selective agonists at the concentration of 50 µmol/L. To further examine the specificity of *theonellasterol* we have also investigated its effects on the expression of several nuclear receptors and co-regulatory factors by a microarray analysis. Results of this experiment demonstrated that *theonellasterol* itself had no effect on the expression of any of these regulatory factors (not shown) and, as demonstrated in Figure 4E, the sponge steroid caused no changes in the expression of these nuclear receptors and regulatory factors in cells challenged with CDCA.

### Effects of FXR antagonism on liver injury induced by BDL in mice

**Figure 5:**
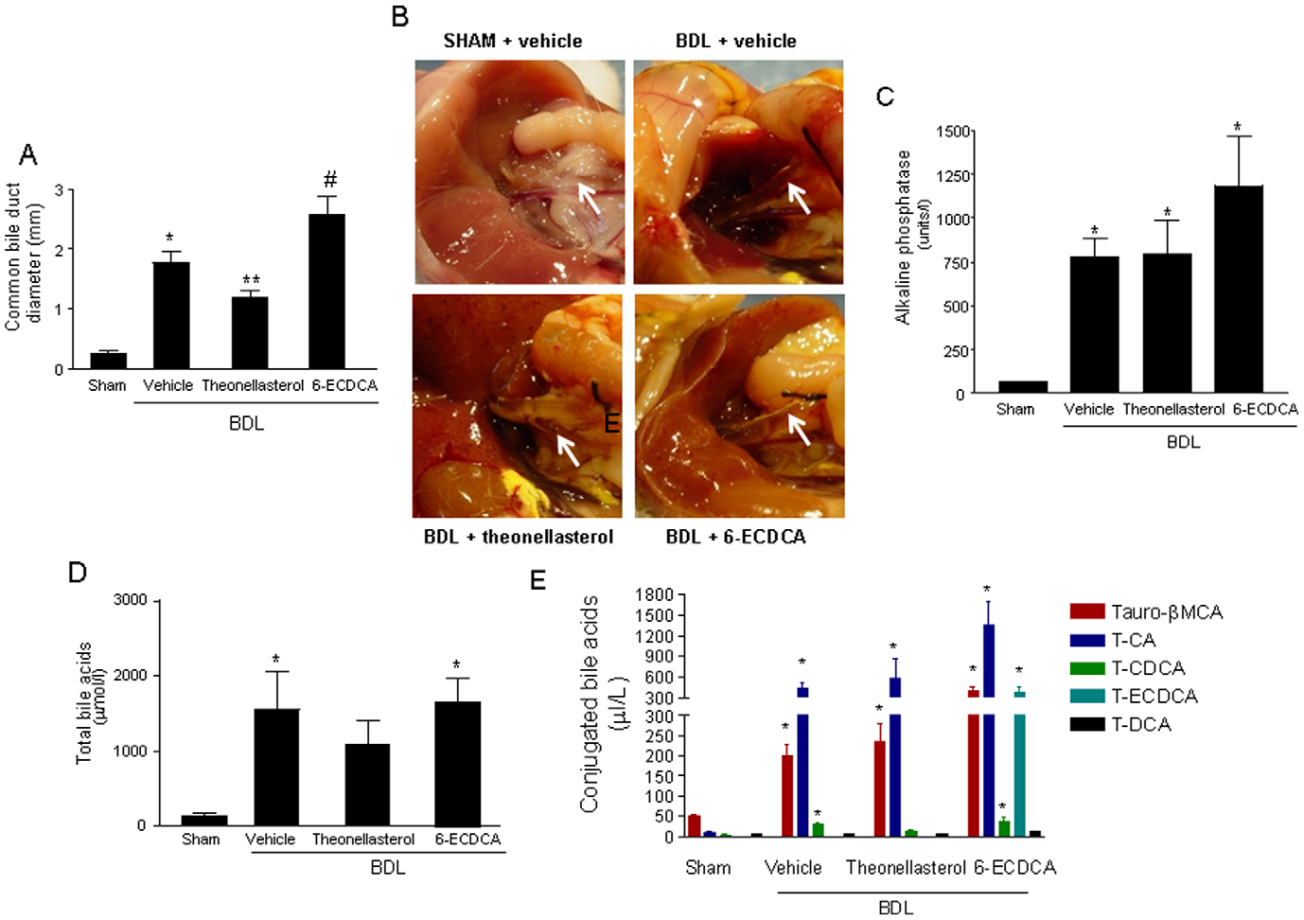
FXR agonism and antagonism lead to development of different patterns of liver injury in response to BDL. (*A*) Common bile duct dilation, 3 days after BDL, is worsened by 6-ECDCA and attenuated by *theonellasterol*. Data are mean ± SE of 6 mice per group. *P<0.05 versus sham operated. ** Theonellasterol versus BDL. (B) Representative macroscopic features of common bile duct (white arrows) in 3-day BDL mice administered *theonellasterol* and 6-ECDCA . (C) BDL increases serum levels of alkaline phosphatase, a marker of bile duct obstruction. Serum levels of alkaline phosphatase were not changed by administering BDL mice with *theonellasterol*, while were slightly increased by 6-ECDCA. Data are the mean ± S.E. of 6 mice per group. *P<0.05 versus sham operated. (D) Serum concentration of total bile acids. Data are the mean ± S.E. of 6 mice per group. *P<0.05 versus sham operated. (E) Quantitative analysis of tauro-conjugated bile acids in BDL animals. BDL increases serum concentrations of T-CA, T-MCA and T-CDCA while secondary bile acids were almost undetected. Challenging BDL mice with 6-ECDCA increases serum levels of T-6-ECDCA. Data are the mean ± S.E. of 4 mice per group. *P<0.05 versus sham operated.

Because these data illustrate that *theonellasterol* is a selective FXR-antagonist, we have designed a proof-of-concept study to ascertain whether this compound was effective in attenuating liver injury caused by BDL in mice, a model of obstructive cholestasis that is attenuated by FXR gene ablation [3]. For this purpose BDL mice were administered the *theonellasterol*, 10 mg/kg, or an FXR agonist, 6-ECDCA, 30 mg/kg, for 3 days. As shown in Figure 5A and B, 3 days after BDL the size of the common bile duct increased ∼10 fold (Figure 5A and B, arrows) in comparison to sham operated animals, and while this effect was exacerbated by administering mice with 6-ECDCA, it was attenuated by the *theonellasterol*, suggesting a direct role for FXR in activation of hepatocyte's apical bile acid output in this setting. Changes in the common bile duct size caused by BDL were not explained by any reduction in the severity of bile duct obstruction, since the serum concentrations of alkaline phosphatase (Alk. Phosp.), a marker of bile duct obstruction were unchanged by *theonellasterol* administration, but slightly increased by 6-ECDCA (Figure 5C). The serum concentrations of total bile acids increased dramatically under condition of BDL, and these changes were partially attenuated by *theonellasterol* but not by the FXR agonist ( Figure 5D and E) . Quantitative analysis of taurine (T)-conjugated bile acids (Figure 5E) and unconjugated bile acids (Figure S4) by liquid chromatography-mass spectrometry demonstrated that BDL caused a marked increase in serum concentrations of T-CA) and T-βmuricolic acid (T-βMCA). Pharmacological interventions on BDL mice had minor impact on serum bile acid concentrations/composition, thought that administering mice with 6-ECDCA resulted in a significant accumulation of its T-ECDCA in the blood (Figure 5E).

**Figure 6:**
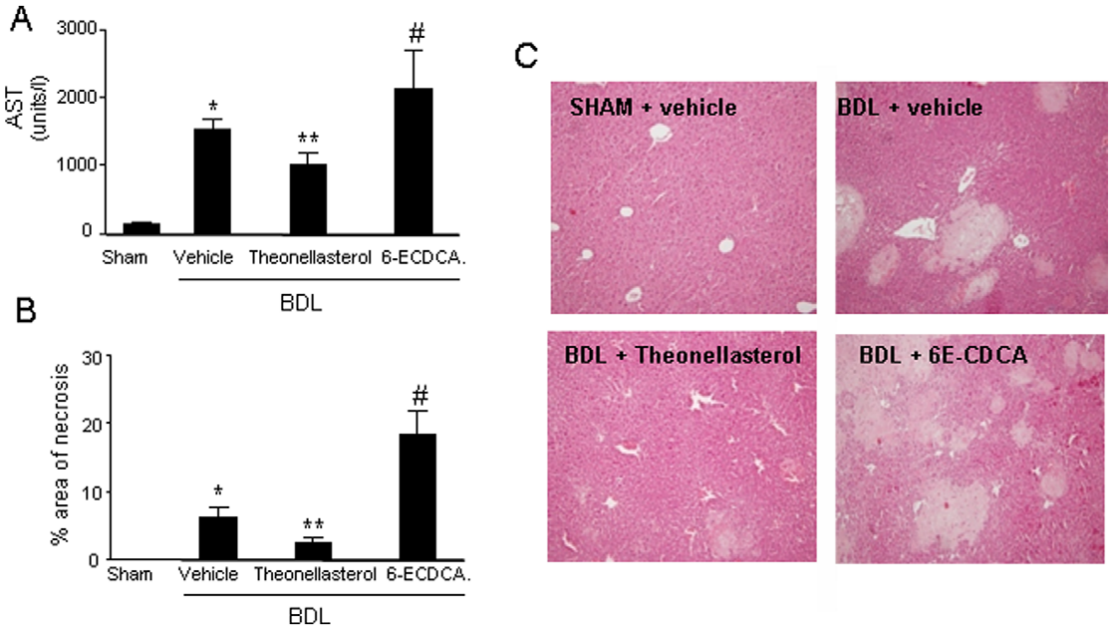
BDL causes liver cell injury and theonellasterol attenuates liver necrosis. (A and B) Liver necrosis was robustly attenuated by *theonellasterol* as confirmed by assessment of ALT and histopathology analysis. Data are the mean ± S.E. of 6 mice per group. *P<0.05 versus sham. **P<0.05 versus BDL. #P<0.05 versus BDL plus *theonellasterol*. (C) Representative liver histology from an individual mice per group. Liver sections were stained with H&E, original magnification 10×.

Liver injury was examined by measuring serum ALT concentrations. Serum ALT values were markedly increased in BDL mice, but significantly reduced by theonellasterol, while the opposite was observed in animals exposed to 6-ECDCA (Figure 6A; n = 6; P<0.05 BDL versus sham operated and *theonellasterol* versus BDL alone). Changes in AST levels were consistent with the histopathology analysis. BDL causes hepatocyte's death that is secondary to intrahepatic bile acid overload. These necrotic areas are confluent foci of hepatocyte feathery degeneration due to bile acid cytotoxicity. The extent of these necrotic foci in the liver was assessed by conventional H&E staining and quantitated using digital image analysis. Histopathological examination of liver specimens demonstrated numerous necrotic foci in untreated BDL mice. This pattern was attenuated by *theonellasterol* treatment as also confirmed by histometric analysis of the extent of liver necrosis (Figure 6B and C; P<0.05 *theonellasterol* versus BDL alone). The opposite was observed in BDL mice administered 6-ECDCA.

**Figure 7:**
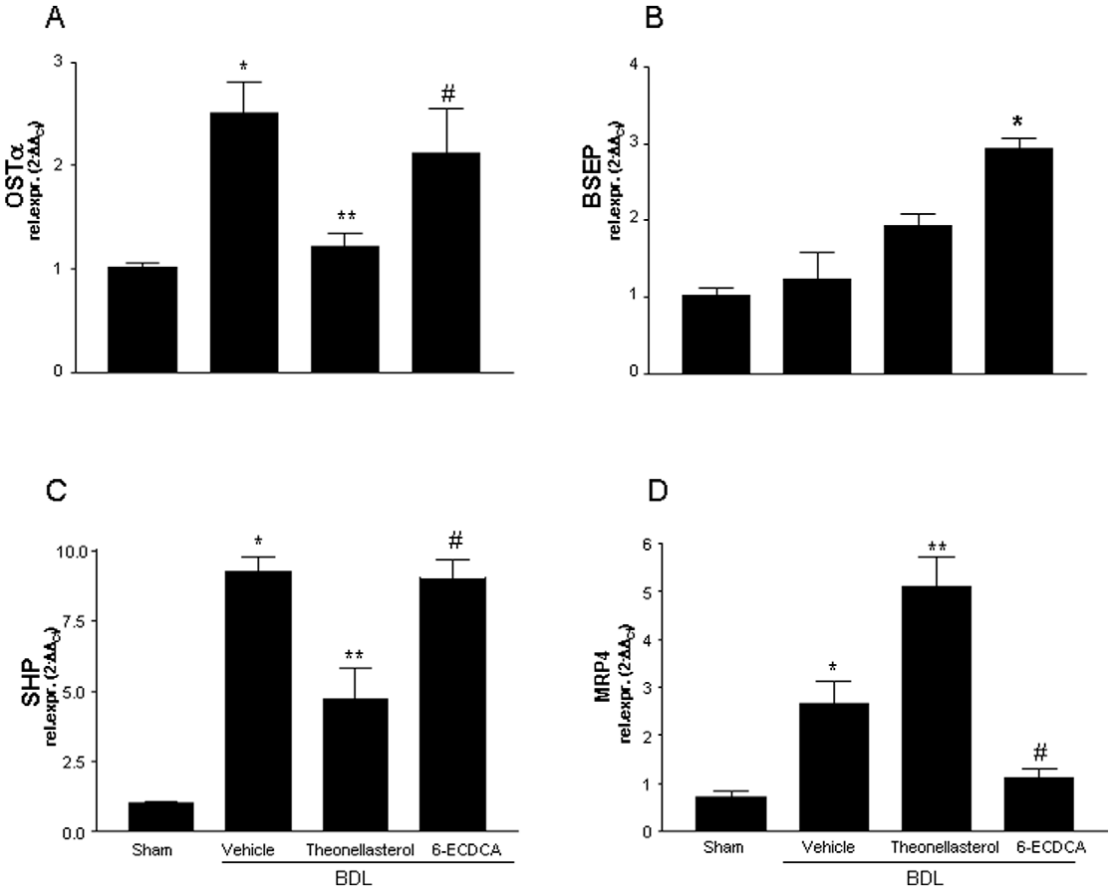
*Theonellasterol* alters the liver expression of genes involved in bile acid metabolism in BDL mice. (A) Real-Time PCR analysis of OSTα, (B) BSEP, (C) SHP and (D) MRP4. Data are the mean ± S.E. of 4 mice per group. *P<0.05 versus sham. **P<0.05 versus BDL. #P<0.05 versus BDL plus *theonellasterol*.

Analysis of the expression of genes involved in bile acids handling by the liver revealed that BDL upregulated the expression of OSTα, MRP-4 and SHP mRNAs (Figure 7A–D; n = 4–6; P<0.05 versus BDL alone). This pattern was dramatically changed by administering mice with *theonellastero*l: in fact the marine steroid reversed the induction of OSTα and SHP caused by BDL and caused a robust induction in MRP-4 mRNA expression (Figure 7; n = 4–6; P<0.05 versus BDL alone). The opposite was observed in mice administered 6-ECDCA, since this FXR agonist reduced the expression of MRP-4 in comparison to BDL alone (n = 4–6; P<0.05 versus BDL alone). Finally, while BDL *per se* had no effect on BSEP mRNA, the expression of this transporter was slightly induced by treating mice with the FXR agonist (Figure 7; n = 4–6; P<0.05 versus BDL).

## Discussion

The pharmaceutical research has expanded in the last two decades to include massive screening of marine organisms in the search of novel drug candidates [9]. Among marine organisms, sponges of the genus *Theonella* have attracted a great interest from the scientific community for the impressive variety of bioactive secondary metabolites with unusual structures and powerful biological activity. In the last year extensive decoding of interaction of *Thenonella swinhoei* sterols with mammalian targets has allowed the identification of a number of marine steroids that function as putative ligands for two nuclear receptors, FXR and PXR [13]. Despite sponges are the simplest animal organisms, phylogenetic analyses have revealed the existence of an ancestral nuclear receptor that existed near the base of the *Metazoa* with fatty acids as possible ancestral ligands [24]. Indeed, analysis of sequenced genome of the demosponge *Amphimedon queenslandica* has shown the presence of sequences encoding for two nuclear receptors, named AqNR1 and AqNR2 [24]. The phylogenetic analysis indicates that AqNR2 is orthologous to hepatocytes nuclear factor (HNF)4, while AqNR1 is the unduplicated ortholog of all other nuclear receptors which subsequently gave rise to all other classes found in vertebrates. Because AqNR1 is the precursor of all modern nuclear receptors it is likely that its ligand binding domain accommodates a variety of structurally different steroids, making sense to the extraordinary biodiversity of marine steroids. The present discovery suggests that in addition to agonists of nuclear receptors, marine sponges could generate steroids endowed with antagonistic activity raising the question of the functional roles these steroids might exert in the context of sponge biology.

In the present study we report that *theonellasterol* is a non-promiscuous antagonist for FXR [21]–[23]. This specificity was confirmed by transactivation and microarray experiments. Indeed, exposure of HepG2 cells to this agent, while abrogates the transactivation of FXR caused by its natural ligand CDCA, fails to modulate the expression of a wide array of mammalian nuclear receptors and fails to activate PPARγ, LXR, PXR and VDR in transactivation assay. These results were confirmed by analysis of expression of genes that are directly regulated by FXR. Thus the *theonellasterol* antagonizes the effects of CDCA on expression of OSTα, BSEP and SHP mRNAs. Because FXR regulates the expression of these genes by direct binding to an FXR responsive element [2], [23] in their promoter, these data provide a robust support to the notion that *theonellasterol* is a selective FXR antagonist. Further confirmation to this view was obtained by the analysis of the effect that *theonellasterol* exerts on the expression of MRP-4. We have previously demonstrated that FXR binds to an overlapping site in the MRP-4 promoter thus causing the displacement of CAR [5]. Because transcription of the MRP-4 gene is under the negative control of nuclear corepressor NCoR, binding of CAR to its consensus element displaces NCoR from the MRP-4 promoter, while FXR causes the reverse [2], [5]. Because these data predict that exposure to an FXR agonist would reverse the negative effects of FXR on MRP-4 in obstructive cholestasis, we have examined whether *theonellasterol* attenuates binding of FXR to the MRP-4 promoter in the presence of CDCA. Indeed, the results of this experiment, shown in Figure 3E, demonstrate that the counter-regulatory effect of CDCA on MRP-4 gene expression is robustly reverted by *theonellasterol*. Because hijacking of a CAR regulated mechanism by FXR agonists, represses MRP-4 gene expression and contributes to bile acid-mediated liver injury in cholestasis, its reversal by an FXR antagonist might hold utility in treatment of these disorders [2].

*Theonellasterol* is significantly different from another natural FXR antagonist, guggulsterone [21]. Indeed, while this agent was originally reported to act as a FXR antagonist, further studies have shown that it functions as a ligand for multiple nuclear receptors [21], [25]–[28], including PXR, the GR, the androgen and mineral corticoid and progesterone receptors. Taking into consideration that agonism of guggulsterone for these receptors takes place at concentrations that are 20–125 lower than that required for FXR antagonism, it is now widely accepted that agonism to these nuclear receptors, rather than FXR antagonism, explains the pharmacological effects of this plant steroid [23], [25]–[28].

In the last decade, FXR has emerged as an important therapeutic target in the treatment of liver disorders [1], [2], [7], [23]. Among other conditions, FXR agonists have been proposed as potential treatment for cholestatic liver disorders, i.e. a spectrum of hepatobiliary diseases of diverse etiologies that are characterized by impaired hepatocellular secretion of bile, resulting in accumulation of bile acids, bilirubin, and cholesterol in hepatocytes [1], [2]. Hepatic defence against bile acid overload involves the activation of a complex network of metabolic pathways leading to the repression of bile acid uptake at the hepatic basolateral membrane of hepatocytes, inhibition of *de novo* synthesis of these end product of cholesterol as well as induction of alternative basolateral bile acid transporters [2]. A number of genes encoding for basolateral and apical transporters undergo adaptive changes in cholestasis. These changes are orchestrated by modulation by a network of transcription factors including the hepatocyte nuclear factors (HNF1, 3, 4), FXR, PXR, CAR , the liver receptor homologue-1 (LRH-1), SHP and the glucocorticoid receptor [1], [2], [23], [29]. The functional relevance of these regulatory elements in orchestrating adaptive responses has been highlighted by various knockout models. Thus, mice lacking PXR or CAR are more susceptible to bile acid induced liver injury than their wild-type littermates [6], [25], [29], [30]. In contrast, FXR gene ablation leads to protection against liver injury induced by BDL [3]. This protection is the result of lower serum bile acid concentrations and altered expression of hepatic transporters, including BSEP, MDR-1 and MDR-2 and MRP-4 [3]. Of interest, in comparison to wild type mice, FXR^−/−^ mice adapt to bile duct ligation by a marked up-regulation of MRP-4 [3]. Despite these observations predict that the use of an FXR antagonist should be protective in the context of obstructive cholestasis, the testing of this hypothesis has been precluded by the lack of a selective FXR antagonist. The discovery that *theonellasterol* is a selective FXR antagonist has allowed us to carry on a proof-of-concept study in BDL mice. The results of this study demonstrated that *in vivo* administration of *theonellasterol* effectively reduced intrahepatic bile duct pressure, as assessed by measuring the size of common bile duct, and reduced the extent of liver injury, as assessed by AST plasma levels and liver histopathology, in BDL mice. Further on, analysis of expression of MRP-4 in the liver of BDL mice, confirmed that pharmacological antagonism to FXR has the potential to increase MRP-4 gene expression while the opposite was observed in mice administered with a potent FXR agonist.

Present results obtained by administering mice with a FXR agonist are partially consistent with previous observations made in the BDL model in rats. Indeed, we have previously shown that long term administration of 6-ECDCA worsened the severity of cholestasis as assessed by measuring alkaline phosphatase and γGT [31]. In contrast, short term administration of GW4064, a non steroidal FXR agonist, has been reported to attenuate liver injury in BDL rats [32]. While the different chemical structure of these ligands might provide an explanation for these findings, the role of FXR agonism in obstructive cholestasis still await confirmation [1], [7].

In summary, we have identified a marine steroid that acts as a selective FXR antagonist. Using this agent we have designed a proof-of-concept study in BDL mice whose results provide evidence that pharmacological antagonism for FXR holds potential in the treatment of obstructive cholestasis. Present data might have a translational readout. Indeed, beside biliary stones and tumors, obstructive cholestasis due to inflammation and loss of intra-hepatic bile ducts, is the key feature of advanced, stages III and IV, PBC and PSC. This study paves the way to the development of FXR antagonists for treatment of obstructive cholestasis.

## Materials and Methods

### Sponge material and isolation of theonellasterol

*Theonella swinhoei* (order Lithistida, family Theonellidae) was collected on the barrier reef of Vangunu Island, Solomon Islands, in July 2004. Authorization to collection and exportation of sponge samples was released by Fisheries Department of Solomon Islands Government to IRD (Institut de Recherche pour le Dèveloppement, Polynesian Research Center on Island Biodiversity, BP529, 98713 Papeete, Tahiti, French Polynesia) in the frame of a research project entitled: “Coral Reef Initiative in The South Pacific” (CRISP) ‘Biodiversité et substances marines actives volet Molécules actives soutenu par l’Agence Française pour le Développement”, authorization IRD–AFDCZZ3012-02U. The sponge lyophilized material was kindly provided by Dr. Cecile Debitus, IRD. The sponge is not on the endangered and protected species list (CITES list, www.cites.org) and no specific permits were required for the described field studies.

The sample was frozen immediately after collection and lyophilized to yield 600 g (dry mass) of R3170. Taxonomic identification was performed by Dr John Hooper of Queensland Museum, Brisbane, Australia, where specimens are deposited under the accession number G3122662.

The lyophilized material (600 g) was extracted with methanol (3×2.7 l) at room temperature and the methanolic extract, taken to dryness, was subjected to a modified Kupchan's partitioning procedure as follows. The methanol extract was dissolved in a mixture of MeOH/H_2_O containing 10% H_2_O and partitioned against *n*-hexane (19.7 g). The water content (% v/v) of the MeOH extract was adjusted to 30% and partitioned against CHCl_3_ (17.8 g). The aqueous phase was concentrated to remove MeOH and then extracted with *n*-BuOH (10 g).

The hexane extract was chromatographed by silica gel MPLC using a solvent gradient system from CH_2_Cl_2_ to CHCl_2_∶MeOH 1∶1.

Fraction eluted with CH_2_Cl_2_∶MeOH 99∶1 (351 mg) was further purified by HPLC on a Nucleodur 100-5 C18 (5 µm; 10 mm i.d.×250 mm) with MeOH∶H_2_O (998∶2) as eluent (flow rate 5 ml/min) to give 40.5 mg of theonellasterol (t_R_ = 19.6 min) (Figure S2).

Identity of theonellasterol was established by comparison of its RM (Table S1 and Figure S1) and mass data (Figure S2) with those previously reported [14].

6-ECDCA was synthesized by Dr. Angela Zampella by a modification of previously published methods [8] and as described previously [7]. Identity of the compound was assessed according its RM and mass data (Figure S3) with those previously published [7]. The taurine conjugated derivative of 6-ECDA (T-ECDCA) was synthesized according to methods reported in the supporting information to this paper (Scheme S1) and its identity confirmed by RM and mass analysis (Figure S5).

### Computational Details

We performed the molecular docking calculations by Autodock 4.2 software [16] on quad-core Intel® Xeon® 3.4 GHz, using a grid box size of 94×96×68, with spacing of 0.375 Å between the grid points, and centered at 20.689 (x), 39.478 (y), 10.921 (z), covering the active site of the FXRs [15]–[20]. To achieve a representative conformational space during the docking studies and for taking into account the variable number of active torsions, 10 calculations consisting of 256 runs were performed, obtaining 2560 structures for the sterol **1**. The Lamarckian genetic algorithm (LGA) was employed for docking calculations, choosing an initial population of 600 randomly placed individuals. The maximum number of energy evaluations and of generations was set up to 5×10^6^ and to 6×10^6^ respectively. Results differing by less than 3.5 Å in positional root-mean-square deviation (RMSD) were clustered together and represented by the most favorable free energy of binding. Illustrations of the 3D models were generated the Python software [33].

### Cell culture, transfection, plasmids and Luciferase assays

HepG2 cells (ATCC-LCG collections, Milan Italy) were cultured at 37°C in Minimum Essential Medium with Earl's salts containing 10% fetal bovine serum (FBS), 1% L-glutamine and 1% penicillin/streptomycin as described previously [31]. The transfection experiments were performed using Fugene HD (Roche) according to manufactured specifications. HepG2 cells were plated in a 6-well plate at 5×10^5^ cells/well. For FXR mediated transactivation, cells were transfected with 100 ng pSG5-FXR, 100 ng pSG5-RXR, 200 ng pCMV-βgalactosidase and with 500 ng of the reporter vector p(hsp27)-TK-LUC containing the FXR response element IR1 cloned from the promoter of heat shock protein 27 (hsp27). At 48 h post-transfection, cells were stimulated 18 h with 10 µM CDCA, with 10 µM theonellasterol or with the combination CDCA (10 µM) plus theonellasterol (50 µM).

To investigate the theonellasterol specificity, HepG2 cells were transiently transfected with 500 ng reporter vector p(UAS)_5X_TKLuc, 200 ng pCMV-βgalactosidase and with a series of vectors containing the ligand binding domain of various nuclear receptors (PPARγ, VDR and GR) cloned upstream of the GAL4-DNA binding domain (i.e. pSG5-PPARγLBD-GAL4DBD, pSG5-VDRLBD-GAL4DBD and pSG5-GRLBD-GAL4DBD). 48 h post-transfection, cells were stimulated 18 h with the appropriate nuclear receptor agonist or with the agonist plus theonellasterol (50 µM). To investigate the PXR mediated transactivation, HepG2 cells were transfected with 100 ng pSG5-PXR, 100 ng pSG5-RXR, 200 ng pCMV-galactosidase and with 500 ng of the reporter vector containing the PXR target gene promoter (CYP3A4 gene promoter) cloned upstream of the luciferase gene (pCYP3A4promoter-TKLuc). At 48 h post-transfection, cells were stimulated 18 h with 10 µM rifaximin, with theonellasterol (10 µM) or with the combination of rifaximin (10 µM) and theonellasterol (50 µM).

After treatments, cells were lysed in 100 µl diluted reporter lysis buffer (Promega) and 10 µl cellular lysate was assayed for luciferase activity using the Luciferase Assay System (Promega). Luminescence was measured using an automated luminometer. Luciferase activities were normalized for transfection efficiencies by dividing the relative light units by β-galactosidase activity expressed from cells cotransfected with pCMV-βgal.

### Real-Time PCR

Total RNA was isolated from HepG2 using the TRIzol reagent according to the manufacturer's specifications (Invitrogen). One µg RNA was purified of the genomic DNA by DNase I treatment (Invitrogen) and random reverse-transcribed with Superscript II (Invitrogen) in 20 µl reaction volume. Fifty ng template was added to the PCR mixture (final volume 25 µl) containing the following reagents: 0.2 µM of each primer and 12.5 µl of 2× SYBR Green qPCR master mix (Invitrogen). All reactions were performed in triplicate and the thermal cycling conditions were: 2 min at 95°C, followed by 40 cycles of 95°C for 20 sec, 55°C for 20 sec and 72°C for 30 sec in iCycler iQ instrument (Biorad). The relative mRNA expression was calculated and expressed as 2^−(ΔΔCt)^. Primers used for qRT-PCR were: GAPDH: gaaggtgaaggtcggagt and catgggtggaatcatattggaa; MRP4: ggcgaattgttagctgtggt and cagggctgctgagacacata; BSEP: gggccattgtacgagatcctaa and tgcaccgtcttttcactttctg; OSTα: tgttgggccctttccaatac and ggctcccatgttctgctcac; SHP: gctgtctggagtccttctgg and ccaatgatagggcgaaagaagag.

### Chromatin Immunoprecipitation

10×10^6^ serum starved HepG2 cells were left untreated or stimulated with CDCA (10 µM) or with the combination CDCA (10 µM) plus theonellasterol (50 µM) for 1 h. After treatments cells were cross-linked with 1% formaldehyde 10 min at room temperature and then the reaction terminated by the addition of glycine to a final concentration of 125 mM. Cells were washed in ice-cold PBS and lysed with 500 µl ChIP lysis buffer (1% SDS, 10 mM EDTA, and 50 mM Tris-HCl, pH 8) containing 10 µM PMSF and protease inhibitor cocktail (Sigma), sonicated and centrifuged at 13000 rpm 10 min at 4°C. Fifty µl of each supernatant (Input DNA) were reverse-cross-linked by the addition of 150 µl Elution buffer (1% SDS and 0.1 M NaHCO_3_) and 8 µl NaCl 5M and by heating the mixture to 65°C for 4 h. DNA was recovered from Input by proteinase K treatment at 65°C for 1 h followed by phenol/chloroform (1∶1) extraction, ethanol precipitation and dissolved into 50 µl of molecular biology grade water. Thus, Input DNA was spectrophotometrically quantified and 40 µg chromatin was diluted with ChIP dilution buffer (0.01% SDS, 1% Triton-X-100,1.2 mM EDTA pH 8.0, 16.7 mM Tris-HCl pH 8.0, 167 mM NaCl) containing protease inhibitors and 20 µl of ChIP lysis buffer equilibrated Protein A Sepharose (Amersham Bioscience) /Salmon Sperm DNA/1% BSA. After mixing at 4°C for 1 h, the mixtures were centrifuged at 1,000 rpm for 1 min to obtain pre-cleared supernatants. Pre-cleared supernatants were immunoprecipitated overnight at 4°C with 4 µg specific antibodies: anti-FXR (sc-13063 - Santa Cruz, CA) or anti IgG (SA1-36098 Pierce) as negative control. Immunoprecipitates were washed sequentially with low-salt wash buffer (0.1% SDS, 1% Triton-X-100, 2 mM EDTA pH 8.0, 20 mM Tris-HCl pH 8.0, 150 mM NaCl) and then with high-salt wash buffer (0.1%SDS, 1% Triton-X-100, 2 mM EDTA pH 8.0, 20 mM Tris-HCl pH 8.0, 500 mM NaCl). DNA was eluted by addition of 250 µl Elution Buffer and the cross-linking reactions were reversed by heating the mixture to 65°C overnight. The DNA was recovered from immuneprecipitated material by proteinase K treatment at 65°C for 1 h followed by phenol/chloroform (1∶1) extraction, ethanol precipitation and dissolved into 50 µl of molecular biology grade water. Five microliters chromatin was used for quantitative real-time PCR. Raw data analysis was performed as follows: ΔCt was calculated versus the input DNA concentration; ΔΔCt was versus unstimulated cells immunoprecipitated with the anti-IgG antibody (experimental condition set as 1.0); the relative expression was calculated as 2^−(ΔΔCt)^. The sequences of primers used for the amplification of the MRP4 promoter were: ttcctttcccaatctaagggg and gatggatgaatgctgtcgtc.

### Microarray analysis

Total RNA from HepG2 cells left untreated, stimulated with CDCA (10 µM) or with the combination of CDCA and theonellasterol (50 µM) was extracted with Trizol reagent (Invitrogen) and reverse transcribed with Superscript-II reverse transcriptase (Invitrogen) following the manual instructions. 100 ng cDNA was pipetted in each well of a 96 well PCR array plate (Human Nuclear Receptors and Coregulators RT^2^ *Profiler*™ PCR Array - http://www.sabiosciences.com/rt_pcr_product/HTML/PAHS-056A.html - Superarray Bioscience, Frederick, MD, USA) and amplified following the manual instructions. Genes selected for PCR analysis encode several classes of nuclear receptors and co-regulators of transcription, including co-activators and co-repressors. PCR analysis was carried out with the on-line software *RT^2^ Profiler PCR Array Data Analysis* (http://pcrdataanalysis.sabiosciences.com/pcr/arrayanalysis.php).

### Bile Duct Ligation

CD1 mice, 27–30 grams, (Harlan Nossan, Italy) were housed under controlled temperatures (22°C) and photoperiods (12∶12-hour light/dark cycle), allowed unrestricted access to standard mouse chow and tap water and allowed to acclimate to these conditions for at least 5 days before inclusion in an experiment. Protocols were approved by the University of Perugia Animal Care Committee according to the Italian guideline for care and use of laboratory animals. The ID for this project is #98/2010-B. The authorization was released to Prof. Stefano Fiorucci, as a principal investigator, on May 19, 2010. On the day of surgery, mice were anesthetized with isoflurane and aseptically subjected to ligation of the common bile duct. Sham operations were performed by gently touching the common bile duct with forceps. Animals (*n* = 6 per group) were sacrificed by exsanguination under anesthesia 3 after BDL or sham operation. The drugs was administered intraperitoneally (25 mg/kg) for 3 days starting to the end of chirurgical procedure. The sham and BDL group received vehicle (100 µl of physiologic solution containing 10% DMSO and 30% ethanol). The latest day the drugs were administered 4 h before the sacrifice. Mortality and the frequency of complications was less than 30%. At the time of sacrifice, blood samples were collected for determination of aspartate aminotransferase (AST) as a measure of liver injury and the diameter of bile duct measured by computer caliper.

### Assessment of Liver Histology

Consecutive sections of liver (4 µm thick) from paraffin-embedded liver were stained with hematoxylin and eosin. The extension of necrosis was quantified using a computerized image analysis system (ImageJ Acquisition System). Images were acquired with a BX60 microscope (Olympus Co., Rome, Italy) and digitalized using a C14 camera (Diagnostic Instruments Inc., Sterling Heights, MI) with a resolution of 1315×1033 pixels. The extension of necrosis in blinded specimens was measured at a video screen display magnification and expressed as a percentage (the ratio of damaged area per total analyzed field surface). The average of the percentage taken from 15 random fields was used to generate a single data for each liver.

### Bile acids determination

The stock solutions of the individual tauro-conjugated bile acids at a concentration of 1 mg/mL were prepared separately in methanol. All the stock solutions were stored at −20°C. Calibration standards were prepared by combining appropriate volumes of each bile acid stock solution and methanol. The calibration range was from 1 to 10000 nM of each bile acid in the final solution.

Mice serum sample aliquots of 100 µL were deproteinized with 1 mL of cold acetonitrile with 5% of NH_4_OH vortexing for 1 min [34]–[35]. After centrifugation at 16000 g for 10 min, the clear supernatant was transferred to a new vial, snap frozen and lyophilized. The sample was then re-dissolved in methanol–water (2∶1, v/v), centrifuged and transferred into an auto-sampler vial. A bile acids extraction yield of 95% has been measured using bile acids standards addition in plasma sample before and after deproteinization procedure.

### Liquid chromatography and mass spectrometry

For LC–MS/MS analysis, chromatographic separation was carried out on the HPLC–MS system LTQ XL ThermoScientific equipped with Accelera 600 Pump and Accelera AutoSampler system. The mixture was separated on a Jupiter 5 µ C18 column from Phenomenex (150×2.00 mm).

Compounds were separated at a flow rate of 200 µl/min using a methanol–aqueous ammonium acetate (NH_4_OAc) gradient [36]. Mobile phase A (A) was 5% methanol in water containing 2 mM NH_4_OAc at pH 7, mobile phase B (B) was methanol, containing NH_4_OAc at 2 mM. The gradient started at 30% B and increased to 100% B in 20 min, kept at 100% B for 5 min then decreased to 30% B in 1 min and kept at 30% B for 10 min. ESI was performed in negative ion mode, the ion source temperature was set at 280°C. The tune page parameters were automatically optimized injecting taurocholic acid at 1 µM as standard. The MS/MS detection was operated in MRM mode using a collision energy of 20 (arbitrary units), the observed transitions were: Tauromuricholic acid (T-MCA) at 13.5 min MRM of 514.28 Th→514.28 Th, taurocholic acid (T-CA) at 16.6 min MRM of 514.28 Th→514.28 Th, taurochenodeoxycholic acid (T-CDCA) at 18.5 min MRM of 498.29 Th→498.29 Th, taurodeoxycholic acid (T-DCA) at 18.9 min MRM of 498.29 Th→498.29 Th and tauro-6-ethylchenodeoxycholic acid (T-6-ECDCA) at 20.7 min MRM of 526.29 Th→526.29 Th [32].

## Supporting Information

Scheme S1 **Synthesis pathway of Tauro-6-ECDCA.**
(DOC)

Figure S1 **NMR spectra and ITMS spectrum for theonellasterol.**
(DOC)

Figure S2 **HPLC trace for theonellasterol.**
(DOC)

Figure S3 **NMR spectra and ITMS spectrum for 6-ECDCA.**
(DOC)

Figure S4 **Free bile acid concentration in BDL animals.**
(PPT)

Figure S5 **NMR spectra and ITMS spectrum for tauro-6-ECDCA.**
(DOC)

Table S1 **Tabulated NMR data for theonellasterol.**
(DOC)
