# Supplementary material for: Discovery That Theonellasterol a Marine Sponge Sterol Is a Highly Selective FXR Antagonist That Protects against Liver Injury in Cholestasis
Source: PLoS One. 2012 Jan 23;7(1):e30443. doi: 10.1371/journal.pone.0030443 (PMC3264597; doi:10.1371/journal.pone.0030443)
Supplement: Scheme S1 — Synthesis pathway of Tauro-6-ECDCA. (DOC) [file pone.0030443.s001.doc]

Renga et al. Scheme S1

Scheme S1. Synthesis of Tauro-6-ECDCA.

a) Taurine (6 eq.), DMT-MM (3 eq), TEA dry (25 eq), DMF dry; b) AmberliteCG-120, MeOH dry, 80% over two steps.

DMT-MM: 4-(4,6-dimethoxy-1,3,5-triazin-2yl)-4-methylmorpholinium chloride.

TEA: triethylamine

DMF: N,N-dimethylformamide

To a magnetically stirred solution of 6-ECDCA (10 mg, 0.023 mmol) in DMF (2 ml), DMT-MM (19 mg, 0.069 mmol) and triethylamine (80 μl) were added, and the mixture was stirred at room temperature for 10 min. Taurine (17 mg, 0.14 mmol) was then added to the mixture, which was further stirred for 3h. The mixture was concentrated under reduced pressure. To the solid dissolved in methanol (10 mL) was added Amberlite CG 120 sodium form (600 mg). The mixture was stirred for 5 h at room temperature and the resin was removed by filtration, and the filtrate was concentrated and loaded onto a C18 column (3g). Elution with 70% methanol gave the sodium salt of tauro-6-ECDCA in the form of colorless amorphous solid (10 mg, 80%).
