# Supplementary material for: Discovery That Theonellasterol a Marine Sponge Sterol Is a Highly Selective FXR Antagonist That Protects against Liver Injury in Cholestasis
Source: PLoS One. 2012 Jan 23;7(1):e30443. doi: 10.1371/journal.pone.0030443 (PMC3264597; doi:10.1371/journal.pone.0030443)
Supplement: Figure S1 — NMR spectra and ITMS spectrum for theonellasterol. (DOC) [file pone.0030443.s002.doc]

**Renga et al. Figure S1**

1. **^1^H NMR (500 MHz, C_6_D_6_) of theonellasterol**

**
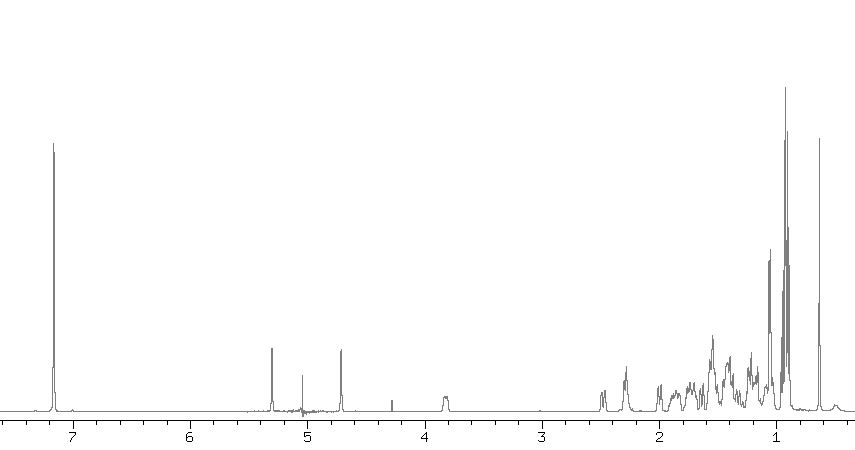
**

1. **^13^C NMR (100 MHz, C_6_D_6_) of theonellasterol**

**
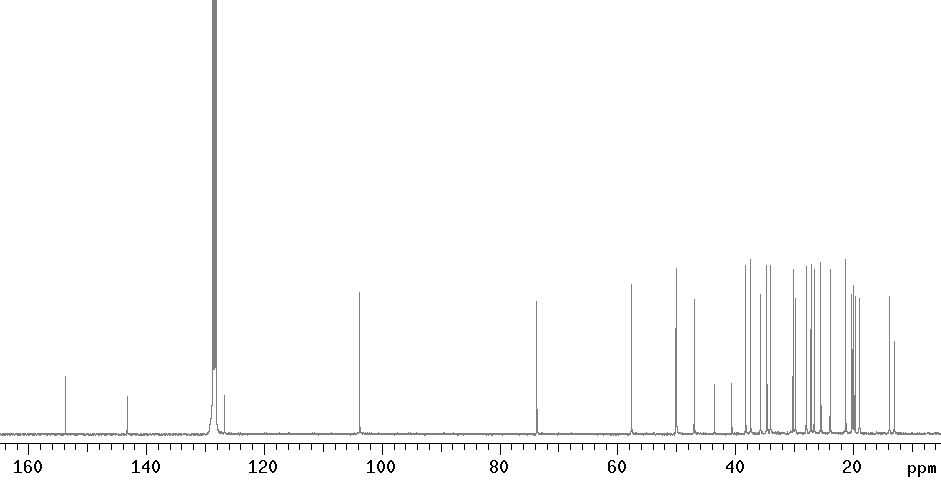
**

1. **COSY spectrum (500 MHz, C_6_D_6_) of theonellasterol**

**
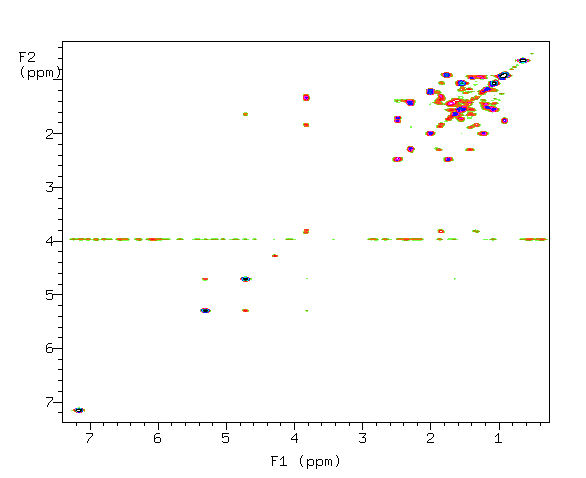
**

1. **HSQC spectrum (500 MHz, C_6_D_6_) of theonellasterol**

**
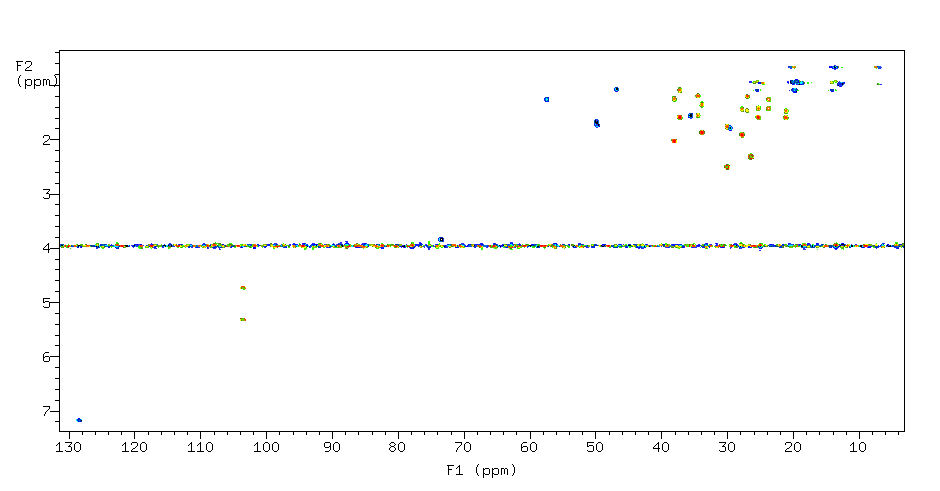
**

1. **HMBC spectrum (500 MHz, C_6_D_6_) of theonellasterol**

**
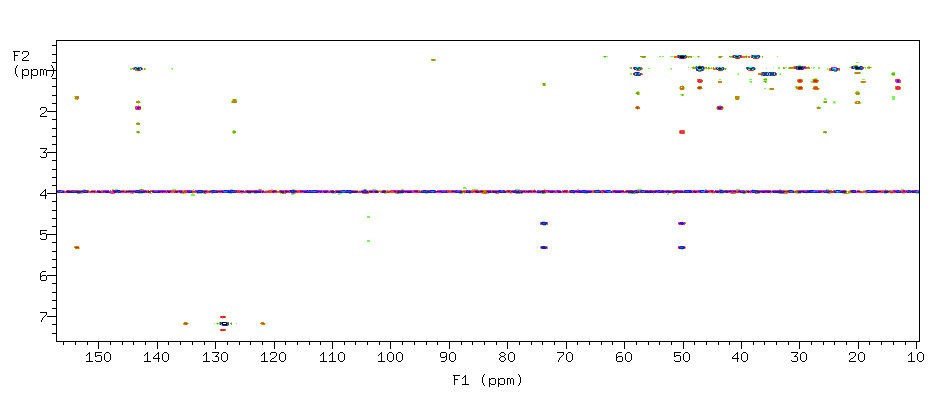
**

**(F) ESI-MS spectrum of theonellasterol**
