# Supplementary material for: Discovery That Theonellasterol a Marine Sponge Sterol Is a Highly Selective FXR Antagonist That Protects against Liver Injury in Cholestasis
Source: PLoS One. 2012 Jan 23;7(1):e30443. doi: 10.1371/journal.pone.0030443 (PMC3264597; doi:10.1371/journal.pone.0030443)
Supplement: Figure S2 — HPLC trace for theonellasterol. (DOC) [file pone.0030443.s003.doc]

**Renga et al. Figure S2**

**Figure S2. HPLC trace of theonellasterol (t_R_=19.6 min) on a Nucleodur 100-5 C18 (5μm; 10 mm i.d. x 250 mm) with MeOH:H_2_O (998:2) as eluent (flow rate 5 mL/min).**

**
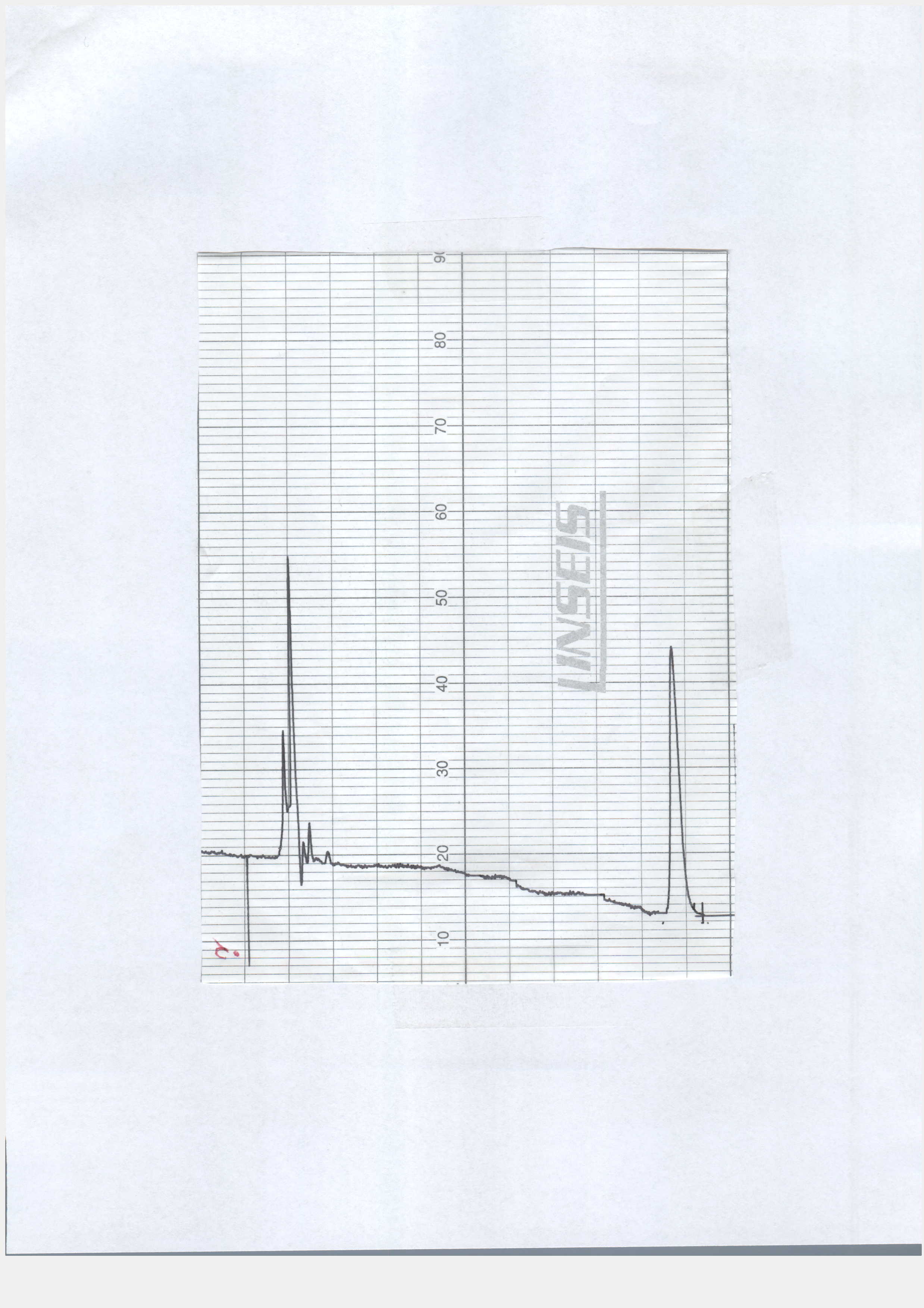
**
