# Supplementary material for: Discovery That Theonellasterol a Marine Sponge Sterol Is a Highly Selective FXR Antagonist That Protects against Liver Injury in Cholestasis
Source: PLoS One. 2012 Jan 23;7(1):e30443. doi: 10.1371/journal.pone.0030443 (PMC3264597; doi:10.1371/journal.pone.0030443)
Supplement: Figure S4 — Free bile acid concentration in BDL animals. (PPT) [file pone.0030443.s005.ppt]

## Slide 1
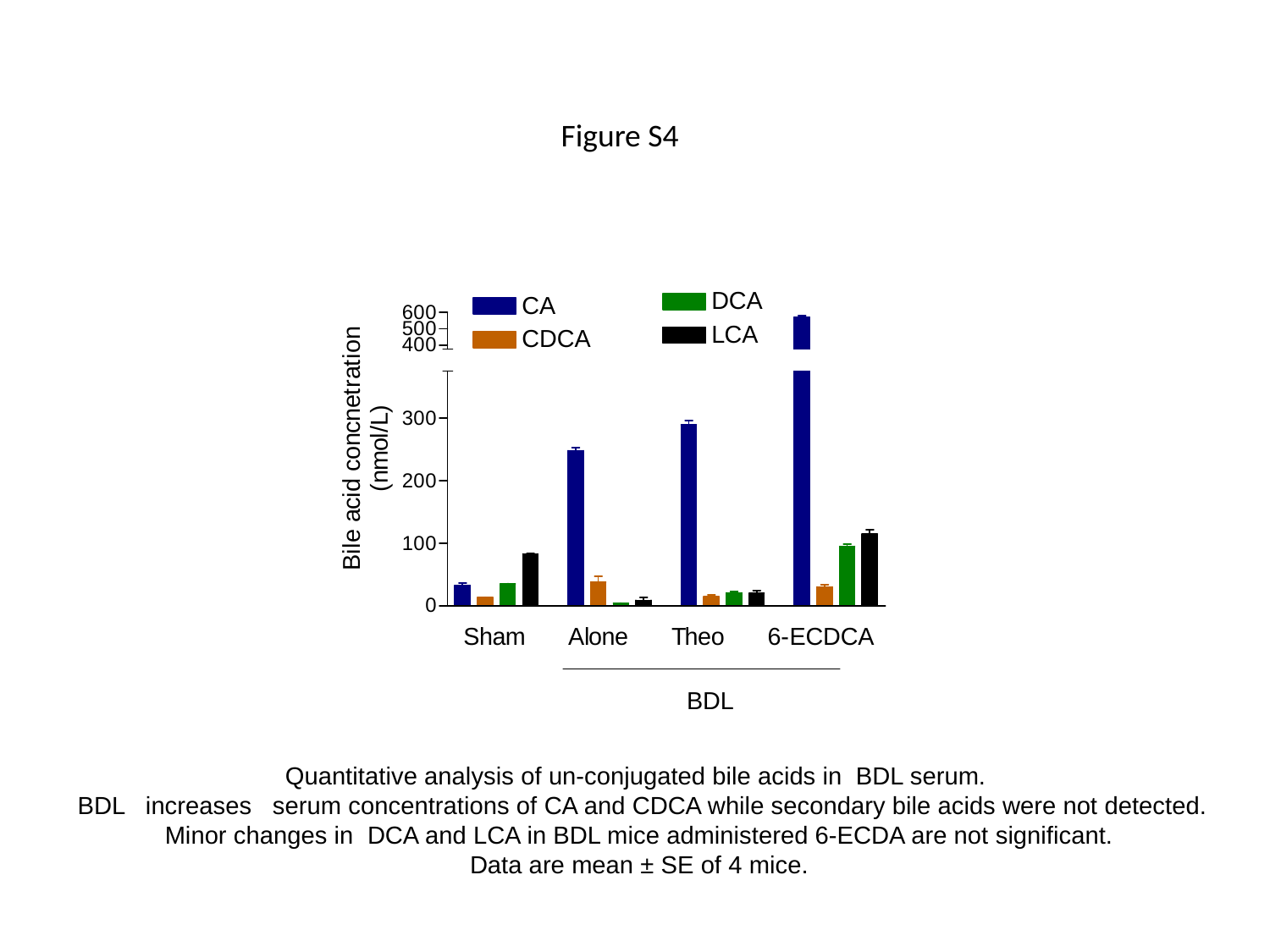

Figure S4
Quantitative analysis of un-conjugated bile acids in BDL serum.
 BDL increases serum concentrations of CA and CDCA while secondary bile acids were not detected.
Minor changes in DCA and LCA in BDL mice administered 6-ECDA are not significant.
Data are mean ± SE of 4 mice.
