# Supplementary material for: Discovery That Theonellasterol a Marine Sponge Sterol Is a Highly Selective FXR Antagonist That Protects against Liver Injury in Cholestasis
Source: PLoS One. 2012 Jan 23;7(1):e30443. doi: 10.1371/journal.pone.0030443 (PMC3264597; doi:10.1371/journal.pone.0030443)
Supplement: Figure S5 — NMR spectra and ITMS spectrum for tauro-6-ECDCA. (DOC) [file pone.0030443.s006.doc]

**Renga et al. Figure S5**

**Figure S5A^. 1^H NMR (500 MHz, CD_3_OD) of Tauro-6-ECDCA**


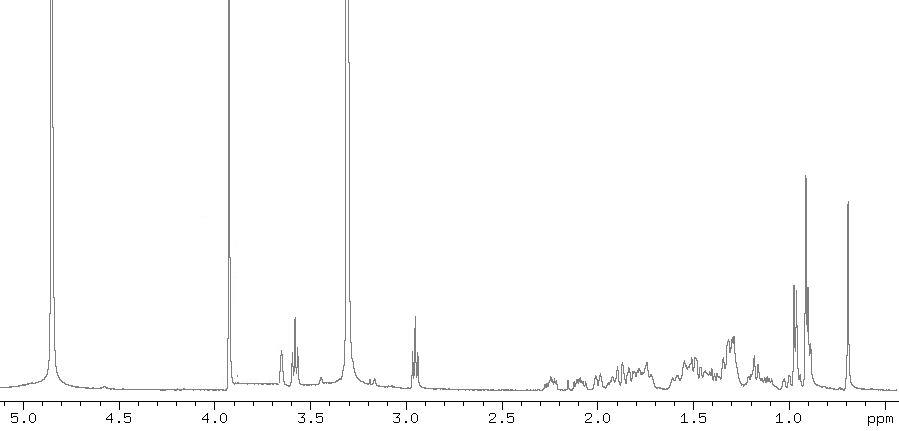


**Figure S5B ^13^C NMR (100 MHz, CD_3_OD) of Tauro-6-ECDCA**


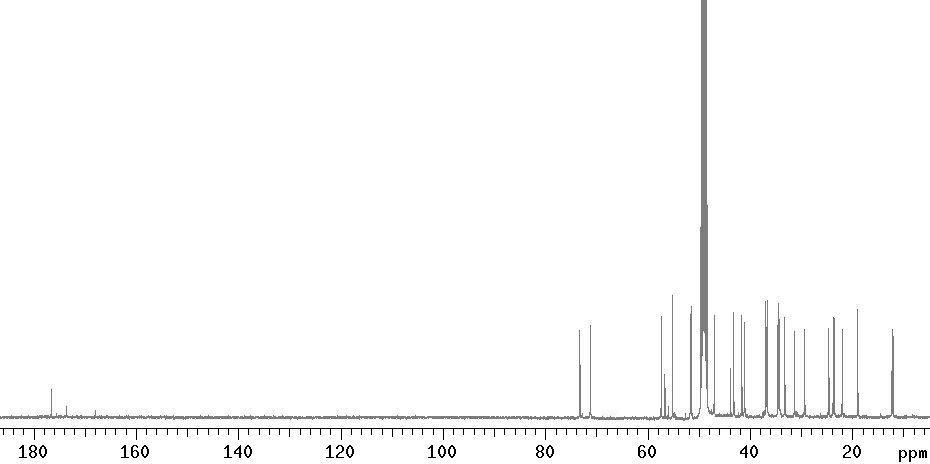


**Figure S5C. ESI-MS spectrum of tauro-6-ECDCA**
