# Supplementary material for: Discovery That Theonellasterol a Marine Sponge Sterol Is a Highly Selective FXR Antagonist That Protects against Liver Injury in Cholestasis
Source: PLoS One. 2012 Jan 23;7(1):e30443. doi: 10.1371/journal.pone.0030443 (PMC3264597; doi:10.1371/journal.pone.0030443)
Supplement: Table S1 — Tabulated NMR data for theonellasterol. (DOC) [file pone.0030443.s007.doc]

Renga et al. Table S1

**Table S1. NMR data (700 MHz, C_6_D_6_) for theonellasterol.**

| position | δ_H_ | δ_C_ | Key HMBC |
| --- | --- | --- | --- |
| 1 | 1.09 m, 1.55 ovl | 37.4 |  |
| 2 | 1.32 ovl, 1.86 m | 34.0 |  |
| 3 | 3.82 m | 73.7 |  |
| 4 | - | 153.9 |  |
| 5 | 2.20 m | 42.6 |  |
| 6 | 1.17 ovl, 1.42 ovl | 27.1 |  |
| 7 | 1.40, 1.90 | 27.9 |  |
| 8 | - | 126.6 |  |
| 9 | 1.64 m | 50.0 |  |
| 10 | - | 40.6 |  |
| 11 | 1.44, 1.54 ovl | 21.2 |  |
| 12 | 1.24 ovl, 2.00 ovl | 38.2 |  |
| 13 | - | 43.5 |  |
| 14 | - | 143.4 |  |
| 15 | 1.42, 1.56 | 25.4 | C13, C14, C17 |
| 16 | 2.29 | 26.6 | C13, C14, C17 |
| 17 | 1.24 ovl | 57.6 | C13, C14, C15 |
| 18 | 0.94 s | 18.9 | C12, C13, C14, C17 |
| 19 | 0.63 s | 13.8 | C1, C5, C9, C10 |
| 20 | 1.53 m | 35.7 |  |
| 21 | 1.06 d (6.4) | 19.9 |  |
| 22 | 1.17 m, 1.53 m | 34.6 |  |
| 23 | 1.75 m, 2.48 m | 30.2 |  |
| 24 | 1.05m | 46.9 |  |
| 25 | 1.77 m | 29.7 |  |
| 26 | 0.90 d (7.0) | 19.6 | C24, C25 |
| 27 | 0.92 d (7.0) | 20.2 | C24, C25 |
| 28 | 1.24 m, 1.40 m | 23.8 |  |
| 29 | 0.95 d (7.3) | 13.0 | C23, C24, C25 |
| 30 | 4.71 br s, 5.30 br s | 103.8 | C3, C4, C5 |

Coupling constants are in parentheses and given in hertz.

^1^H and ^13^C assignments aided by COSY, TOCSY, ROESY, HSQC and HMBC experiments.

Ovl: signals overlapped
